# Supplementary material for: Therapy-Induced Stromal Senescence Promoting Aggressiveness of Prostate and Ovarian Cancer
Source: Cells. 2022 Dec 13;11(24):4026. doi: 10.3390/cells11244026 (PMC9776582; doi:10.3390/cells11244026)
Supplement: Supplementary file 1 [file cells-11-04026-s001.zip › cells-2024728-supplementary.pdf]

Supplementary Figures

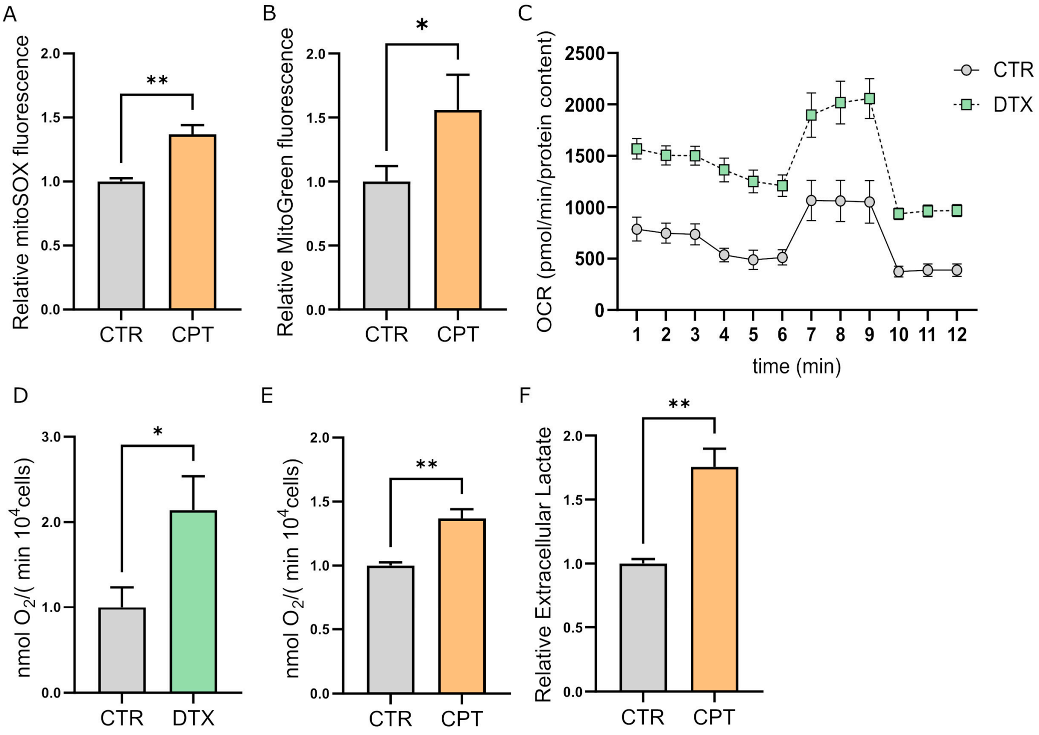

Supplementary Figure S1. Metabolic rewiring in chemotherapy treated fibroblasts. (**A**) Mitochondrial ROS production in CPT-treated fibroblasts. Fibroblasts were stained with MitoSOX and fluorescence was evaluated by FACS analysis. Student's T test (n=4). (**B**) Mitochondrial mass of control or CPT-treated HOFs. Cells were incubated with Mitotracker Green and the mitochondrial mass was measured by FACS analysis. Student's T test (n=3). (**C**) Seahorse XF MitoStress analysis in DTX-treated HPFs. OCR was measured in real time in control and DTX-treated HPFs and normalized on total protein content. ~~(**B**)~~ **(D, E)** Oxygen consumption rate (OCR) of DTX-treated HPFs (D) and CPT-treated HOFs (E) measured with Clarke-O2 type electrode. Student's T test (n=3). **(F ~~D~~)** Extracellular lactate levels quantification in control or CPT-treated HOFs. CM was collected from control or TIS-fibroblasts and clarified by centrifugation. Results were normalized on cell number. Student's T test (n=4). Data are reported as mean ± S.E.M of n independent experiments. $*p < 0.05$; $**p < 0.01$.

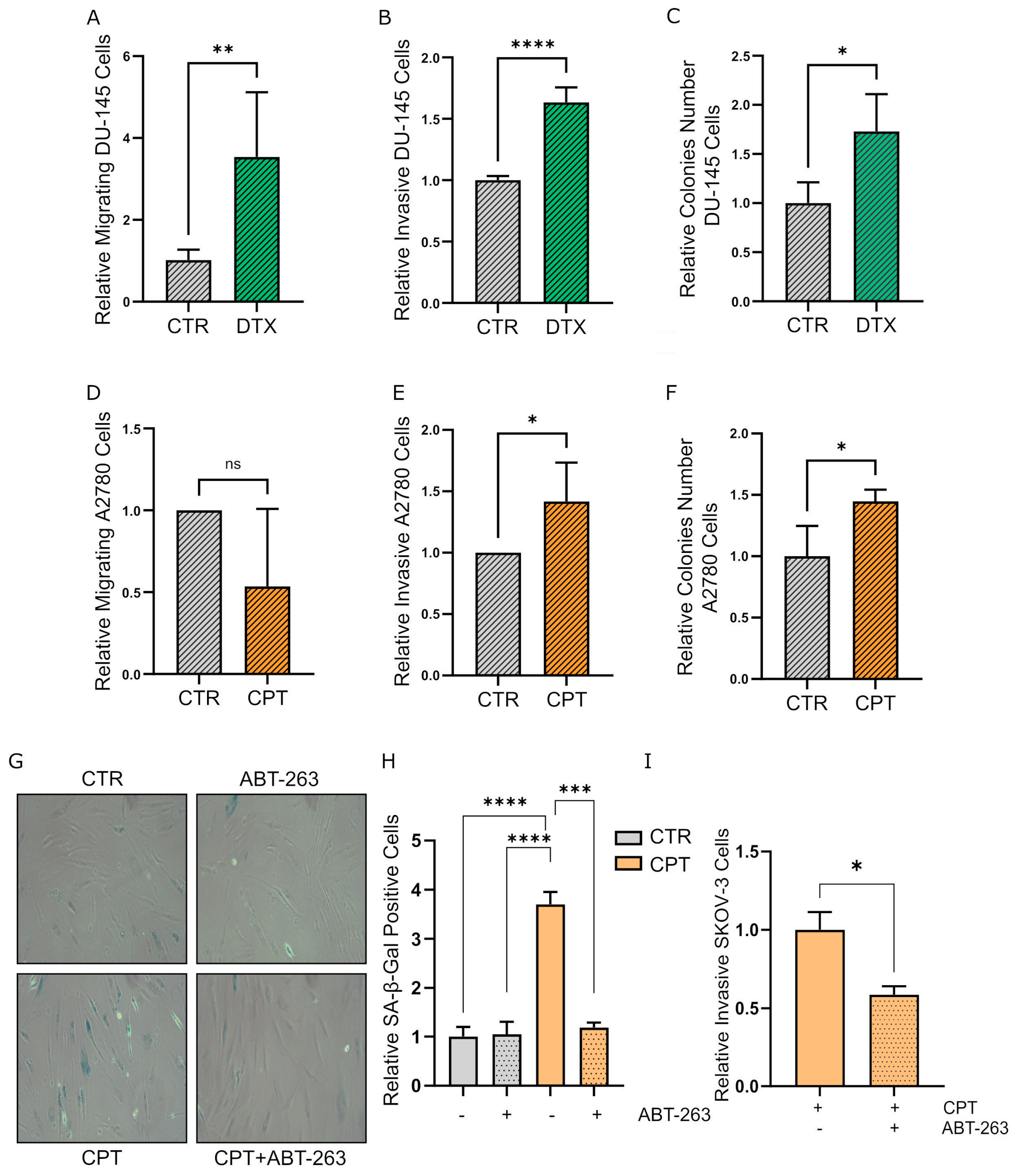

Supplementary Figure S2. TIS prostate and ovarian fibroblasts induce cancer cell aggressiveness. (**A,B; D,E**) Migratory (A, D) and invasive (B, E) abilities of DU-145 (A, B) and A2780 (D, E) cancer cells treated with CM from TIS fibroblasts. Tumor cells were incubated for 72 hours with CM from control or senescent fibroblasts, before being seeded in the upper compartment of 8μm Transwell systems pre-coated (B, E) or not (A, D) with Matrigel. Cells were let to migrate/invade for 16 hours. Migrated/invaded cells were stained with crystal violet and counted (five randomly chosen fields). Student's T test (n ≥3). (**C,F**) Colony formation potential of prostate and ovarian cancer cells. DU145 (C) or A2780 (F) cancer cells were incubated with CM from control and senescent fibroblasts for 72 hours before seeding them in new dishes and incubating for 10 days. Colonies were stained with crystal violet dye, photographed and counted. Student's T test (n=3). (**G**) SA-β-Gal staining of HOFs, following exposure to chemotherapy and the senolytic drug ABT-263. Representative images of control, CPT-treated, ABT263-treated or CPT+ABT263-treated ovarian fibroblasts incubated with SA-β-Gal staining solution are represented. (**H**) Quantification of SA-β-Gal staining in CPT/ABT-263 treated HOFs. For each condition, photos at ten randomly chosen fields were taken and total and blue (SA-β-Gal positive) cells were counted. Data are reported as the relative mean ratio between positive and total cells. One-way ANOVA with Tukey correction (n=3). (**I**) Invasive abilities of SKOV-3 cancer cells incubated with CM from CPT or CPT + senolytic drug treated fibroblasts. After 72 hours of incubation with CM from fibroblasts, SKOV-3 cells were seeded in the upper compartment of 8 μm-Transwell and let to invade toward the lower compartment for 16 hours. Cells were stained with crystal violet and counted (five randomly chosen fields). Student's T test (n=3). Data are reported as mean ± S.E.M of n independent experiments. *$p < 0.05$; **$p < 0.01$; ***$p < 0.001$; ****$p < 0.0001$.
